# Efficacy of Gel Bait Insecticides with Different Modes of Action Against Pyrethroid-Resistant *Blattella germanica* Populations in Türkiye

**DOI:** 10.3390/pathogens15080842

**Published:** 2026-08-13

**Authors:** Burak Polat, Aysegul Cengiz, Samed Koc, Emre Oz, Ozge Tufan-Cetin, Huseyin Cetin

**Affiliations:** 1 Department of Biology, Faculty of Science, Akdeniz University, 07070 Antalya, Türkiye; bpolatant@gmail.com (B.P.); aysegulcengiz@akdeniz.edu.tr (A.C.); 2 Laboratory Animals Application and Research Centre, Akdeniz University, 07070 Antalya, Türkiye; samedkoc@akdeniz.edu.tr; 3 Department of Medical Services and Techniques, Vocational School of Health Services, Antalya Bilim University, 07190 Antalya, Türkiye; emre.oz@antalya.edu.tr; 4 Department of Environmental Protection Technology, Vocational School of Technical Sciences, Akdeniz University, 07070 Antalya, Türkiye; ozgetufan@akdeniz.edu.tr

**Keywords:** *Blattella germanica*, gel bait insecticides, insecticide resistance, neonicotinoids, pyrethroid resistance, urban pest management

## Abstract

The German cockroach, *Blattella germanica* (Linnaeus) (Blattodea: Ectobiidae), is a major urban pest that can cause allergic reactions and mechanically carry pathogenic microorganisms. Its control has become increasingly difficult because of widespread resistance to commonly used insecticides, particularly synthetic pyrethroids. This study compared the efficacy of six commercial gel bait formulations containing imidacloprid, dinotefuran, clothianidin, indoxacarb, spinosad, or fipronil against four field populations of *B. germanica* from Antalya, Türkiye, previously characterized and routinely monitored as pyrethroid-resistant. Adult females, adult males, and nymphs were tested separately, and mortality was recorded at 24 h intervals over a 120 h exposure period. Mortality data were analyzed using one-way ANOVA followed by Duncan’s multiple-range test, while univariate ANOVA was used to evaluate the effects of gel bait formulation, population, developmental stage, and exposure time. Mortality differed significantly among the tested formulations, populations, developmental stages, and exposure times (*p* < 0.001). The dinotefuran-containing formulation produced the highest and most consistent mortality across all populations and developmental stages. The indoxacarb-containing formulation also showed high efficacy, although mortality developed more gradually during the early observation periods. The formulation containing imidacloprid showed the lowest efficacy, whereas those containing clothianidin, spinosad, and fipronil produced intermediate and population-dependent responses. Nymphs were significantly less susceptible than adult females and males. Overall, the efficacy of the tested gel bait formulations was influenced by formulation, field population, developmental stage, and exposure time. These findings provide comparative efficacy data that may support the selection and rotation of gel bait formulations in resistance-aware management programs for *B. germanica*.

## 1. Introduction

The maintenance of healthy and sustainable human living environments relies not only on effective hygiene and sanitation practices but also on the control of biological agents that can contaminate food, surfaces, and indoor environments. Within the One Health framework promoted by the World Health Organization, preventing the transmission of pathogens to humans before the onset of disease is a central public health priority [1]. In this context, integrated preventive strategies targeting organisms that facilitate the spread of pathogenic microorganisms in human habitats are essential for protecting public health. Among these organisms, synanthropic pests play a critical role in the dissemination of pathogens in urban environments [2].

Cockroaches are among the most undesirable pests in human settlements due to their ability to contaminate food and surfaces and to mechanically transmit a wide variety of pathogenic microorganisms [3]. Increasing cockroach populations in urban areas are strongly associated with increased allergic sensitization, asthma exacerbations, and psychological fear associated with the presence of insects [4]. These insects commonly colonize kitchens, food-handling facilities, hospitals, and residential buildings, particularly under conditions of poor sanitation. Among urban pest species, the German cockroach, *Blattella germanica* Linnaeus (Blattodea: Ectobiidae), is one of the most problematic due to its high reproductive capacity, cryptic behavior, and remarkable adaptability. Under favorable conditions, small founder populations can rapidly develop into large infestations, making control efforts particularly challenging [5].

Numerous studies have demonstrated that *B. germanica* acts as a mechanical vector for a wide range of pathogenic microorganisms. Of particular concern, investigations conducted in healthcare settings have reported the presence of multidrug-resistant and clinically significant bacteria in association with German cockroaches, highlighting their potential role in the dissemination of antimicrobial-resistant pathogens [3,6]. Consequently, infestations of *B. germanica* represent not only a nuisance problem but also a significant public health risk, especially in sensitive environments such as hospitals, nursing homes, and food-service establishments. In addition, exposure to cockroach-derived allergens has been strongly associated with increased prevalence and severity of allergic reactions and respiratory disorders [4,7]. These public health concerns underscore the need for effective and sustainable control strategies against *B. germanica* populations.

For decades, the control of the German cockroach has relied primarily on synthetic insecticides, including organophosphates, carbamates, and synthetic pyrethroids, typically applied as residual sprays to harborages and hiding sites [8,9]. However, the extensive and prolonged use of these compounds has led to the widespread development of insecticide resistance in *B. germanica* populations worldwide [10,11]. Resistance to synthetic pyrethroids has been extensively documented and is now recognized as a major factor contributing to control failures in urban pest management programs. These developments have significantly reduced the effectiveness of conventional insecticides and highlighted the urgent need for alternative control strategies with different modes of action.

Insecticide resistance in *B. germanica* is mediated by multiple mechanisms, including enhanced metabolic detoxification, target-site insensitivity, reduced cuticular penetration, and behavioral avoidance, which together substantially reduce the efficacy of conventional contact insecticides [10,11,12]. Metabolic resistance is primarily associated with increased activity of cytochrome P450 monooxygenases, esterases, and glutathione S-transferases, whereas target-site resistance is commonly linked to knockdown resistance mutations in the voltage-gated sodium channel [11,12]. The coexistence of cross-resistance and multiple resistance mechanisms has further limited the effectiveness of traditional chemical control strategies, demonstrating the need for alternative and resistance-aware approaches. In response, insecticidal bait formulations, particularly gel baits, have become a cornerstone of modern German cockroach management. Applied in small quantities to inaccessible locations, gel baits minimize human and non-target exposure while exploiting the foraging behavior of cockroaches [13,14]. In addition to their toxic action, their effectiveness is influenced by factors such as bait palatability, formulation stability, and their capacity to overcome existing resistance mechanisms. Unlike residual insecticides, gel baits can partially circumvent resistance by poisoning insects through ingestion and enabling horizontal transfer within populations. However, despite their widespread use, recent studies have reported reduced efficacy of certain gel formulations, suggesting the development of behavioral or physiological resistance, as well as formulation-related limitations [15]. These limitations highlight the need for comparative evaluations of gel bait formulations with different modes of action, particularly against resistant field populations [11,16]. In Türkiye, published information on insecticide resistance in *B. germanica* remains limited. Available published studies have documented reduced susceptibility of field populations to synthetic pyrethroids and have also reported changes in detoxification enzyme activities associated with insecticide exposure [10,17]. However, comprehensive resistance monitoring data covering different insecticide classes and geographic regions are still lacking, highlighting the need for further evaluations of alternative control products against local field populations. Nevertheless, comparative data on the efficacy of multiple active ingredients against pyrethroid-resistant populations remain scarce, particularly under field-relevant resistance conditions.

Therefore, the objective of the present study was to comparatively evaluate the efficacy of six gel bait formulations with different modes of action against pyrethroid-resistant *B. germanica* populations in Türkiye. Gel baits containing clothianidin, dinotefuran, fipronil, imidacloprid, indoxacarb, and spinosad were assessed to determine differences in performance among active ingredients, populations, and developmental stages based on mortality rates and time to death. This study provides a comprehensive evaluation of gel bait performance under resistance conditions and aims to support evidence-based decision-making in urban pest management while contributing to efforts in reducing public health risks associated with insecticide-resistant cockroach infestations.

## 2. Materials and Methods

### 2.1. Test Insects

German cockroaches, *Blattella germanica* Linnaeus (Blattodea: Ectobiidae), were collected in 2025 from four independent restaurant infestations located in three districts of Antalya, Türkiye. Two geographically separated populations were sampled from Muratpaşa District (designated as Muratpaşa Strain 1: 36.89291° N, 30.70415° E; Muratpaşa Strain 2: 36.889716° N, 30.689205° E), whereas one population each was collected from Kepez (36.9209° N, 30.7286° E) and Konyaaltı (36.8569° N, 30.6289° E). Adult cockroaches and nymphs were collected using a portable handheld vacuum aspirator and transported alive to the laboratory, where field colonies were established. In addition, these populations are routinely monitored by the Akdeniz University Biological Efficacy Test Laboratory, which is authorized by the Turkish Ministry of Health to conduct biological efficacy testing of public health biocidal products. Based on this continuous surveillance, the selected field populations were considered pyrethroid-resistant for the purposes of the present study. These field populations were previously shown to exhibit moderate to high levels of resistance to the synthetic pyrethroids deltamethrin and alpha-cypermethrin [10].

Colonies were maintained under controlled conditions (25 ± 2 °C, 40 ± 10% relative humidity, and a 12:12 h light–dark photoperiod). Cockroaches were reared in 5 L plastic containers and fed a laboratory diet consisting of corn flour, wheat flour, and honey at a ratio of 4:4:1 (*w*/*w*). Water was provided separately via cotton soaked in distilled water. Food and water were replenished at least twice per week. Cylindrical cardboard shelters (e.g., paper towel cores) were placed inside the containers to simulate natural harborages.

For experimental purposes, individuals were separated into three groups and tested independently: nymphs (5–6 weeks old), adult females, and adult males (approximately 1 week old). Adults were sexed prior to the bioassays, and only healthy and active individuals were used in the experiments. Bioassays were conducted using individuals from the F2–F3 generations.

### 2.2. Materials (Description of Gel Bait Formulations)

The gel bait formulations used in this study were commercially available products containing different active ingredients, including clothianidin, dinotefuran, fipronil, imidacloprid, indoxacarb, and spinosad. These active ingredients were selected because they represent different modes of action and/or include the principal active ingredients commonly used in commercial gel bait formulations for German cockroach control in Türkiye [18,19]. To ensure the general applicability of the findings and to avoid potential bias associated with specific commercial products, formulations are presented based on their active ingredients rather than product names.

**Figure 1:**
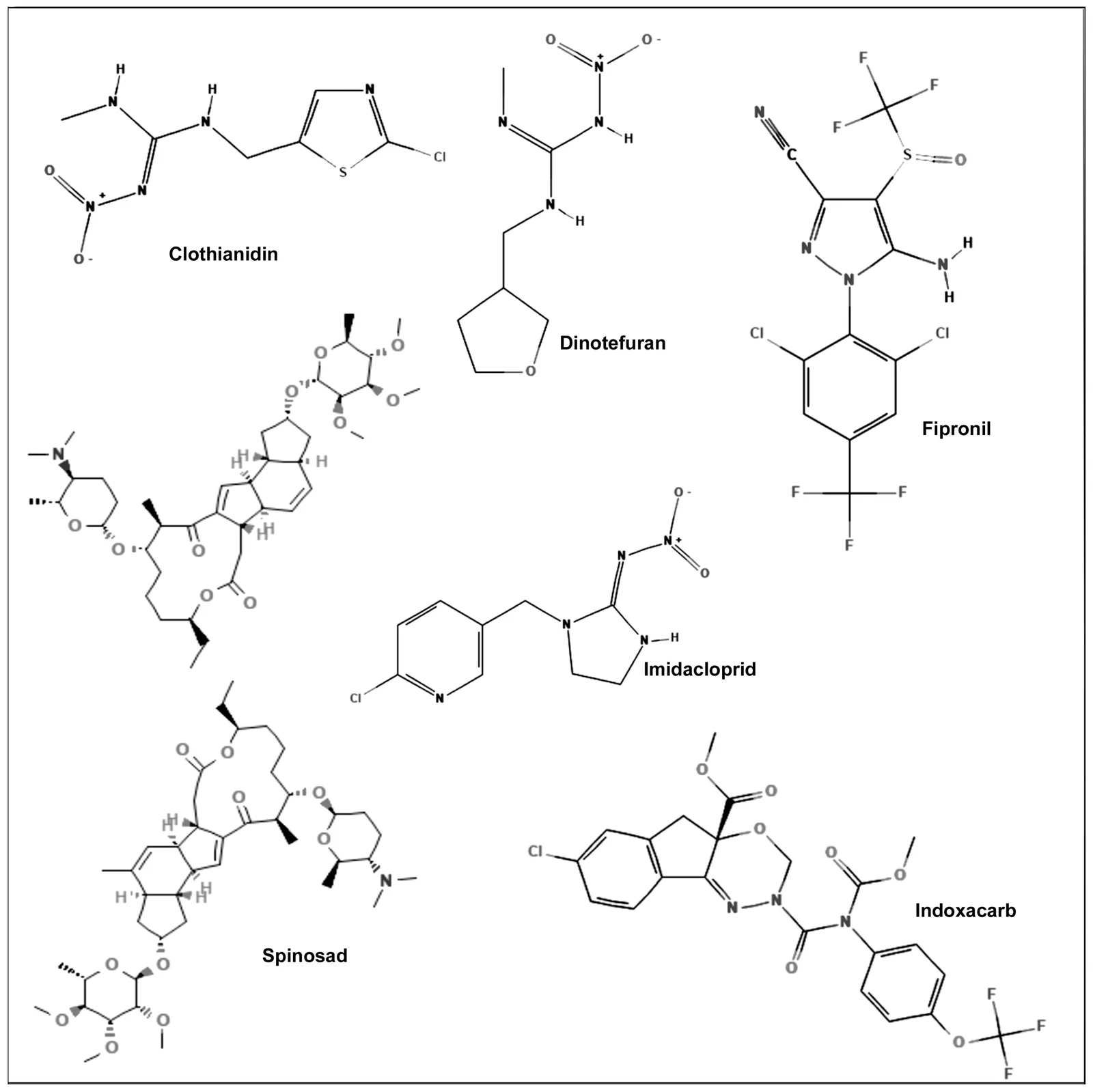
Chemical structures of the six insecticidal active ingredients evaluated in the present study.

The characteristics of the tested formulations, including active ingredients, CAS numbers, concentrations, and modes of action (IRAC classification), are summarized in Table 1. All active ingredients are classified according to the Insecticide Resistance Action Committee (IRAC) mode of action classification. In addition, the chemical structures of all formulations are provided in Figure 1.

### 2.3. Experimental Design

The efficacy of six gel bait formulations with different active ingredients was evaluated against *B. germanica* under laboratory conditions. Bioassays were conducted separately on nymphs (5–6 weeks old), adult females, and adult males (approximately 1 week old). Each treatment was performed using three independent replicates, with 10 individuals housed in a separate glass jar per replicate (*n* = 30 per treatment). Each glass jar was considered one independent experimental unit, and mortality percentages were calculated for each replicate and used in the statistical analyses.

Experiments were conducted using a choice test design, in which cockroaches were allowed to choose between gel bait and an alternative food source. This design was selected to better simulate field conditions and to evaluate feeding-mediated exposure to gel bait formulations. Bioassays were carried out in 1 L glass jars, with the upper 3–5 cm of the inner wall coated with talc (Utalk Pudra, Kurtsan İlaçları A.Ş., Istanbul, Türkiye) to prevent escape.

**Figure 2:**
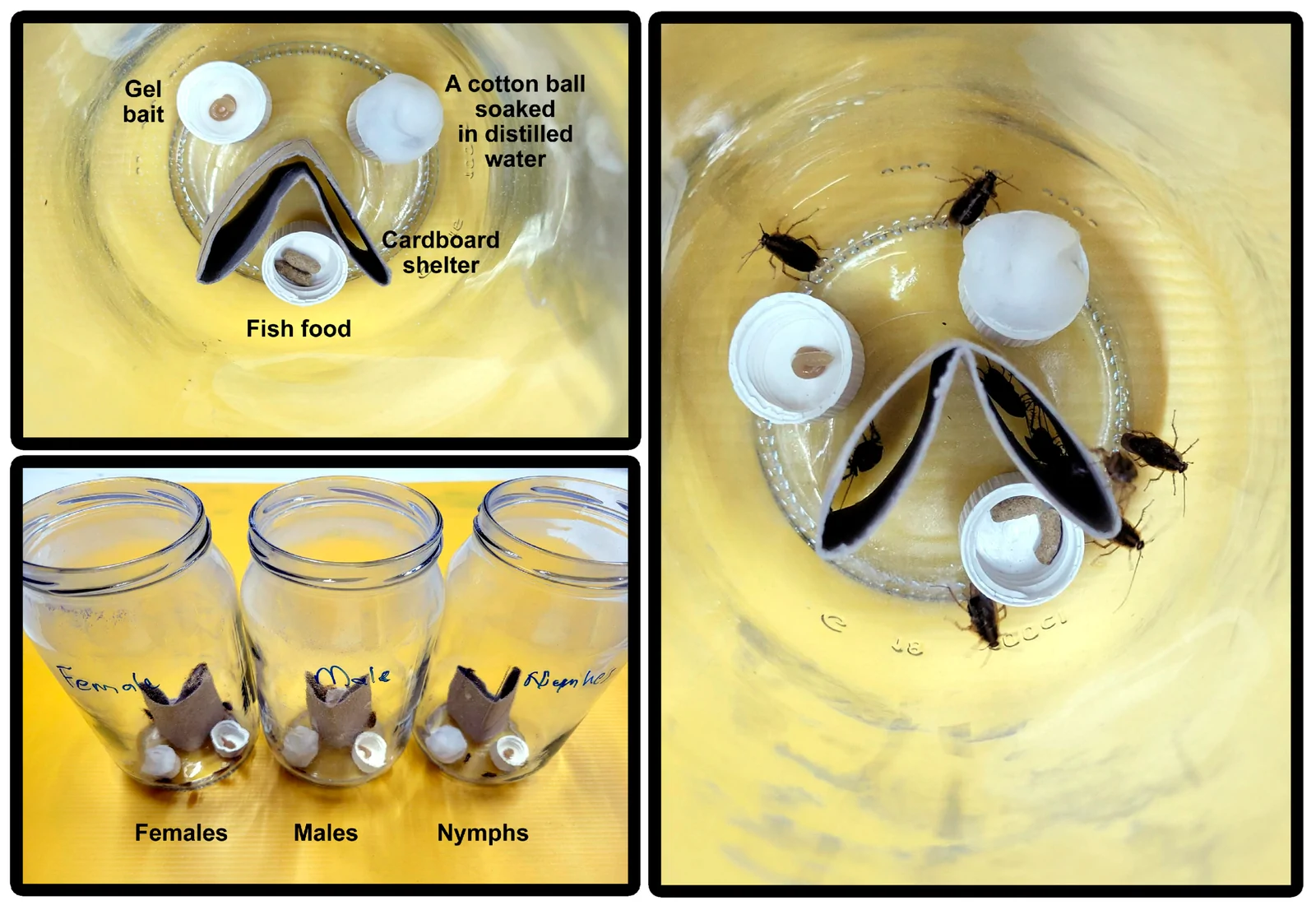
Experimental setup used for laboratory gel bait bioassays.

In each jar, a standardized amount of gel bait (approximately 0.1 g) was applied. In addition, approximately 1 g of commercial fish food was provided as an alternative food source, along with a cotton ball soaked in distilled water to maintain moisture. The gel bait and the alternative food source were placed on the bottom of the jar approximately 3–4 cm apart (Figure 2). Cylindrical cardboard shelters were placed inside the jars to simulate natural harborages. Only one gel bait formulation was tested per container. The positions of the gel bait and the alternative food source were kept constant throughout all experimental replicates. A control group was included under identical experimental conditions. Control jars contained the alternative food source (commercial fish food), water supplied via cotton soaked in distilled water, and a cardboard shelter, but no gel bait.

All experiments were conducted under controlled conditions (25 ± 2 °C, 40 ± 10% relative humidity, and a 12:12 h light–dark photoperiod). Mortality was recorded at 24 h intervals over a period of 5 days (120 h). Individuals were considered dead when no movement was observed after gentle stimulation. Dead individuals were left in the containers throughout the experiment to allow for potential secondary mortality effects.

### 2.4. Statistical Analysis

All statistical analyses were performed using SPSS software (version 20.0; IBM Corp., Armonk, NY, USA). Mortality data are expressed as mean percentage mortality ± standard error (SE). Control mortality was below 5% in all experiments; therefore, no correction using Abbott’s formula was required (Supplementary Dataset S1).

Overall efficacy percentages for each active ingredient were calculated using the mean mortality values recorded at the final observation time (120 h) across all four populations and three developmental stages. Mortality values recorded at earlier exposure times were not included in this calculation.

Prior to statistical analysis, percentage mortality data were subjected to a square root of the arcsine transformation to balance the variance. Using the transformed dataset, mortality differences for nymphs, adult females, and adult males separately and across groups defined by gel formulations and growth types were analyzed using one-way analysis of variance (ANOVA) (*p* < 0.001), followed by Duncan’s multiple-range test (*p* < 0.05) for mean comparisons. For clarity and presentation purposes, untransformed mean values (means ± SE) are reported in the text, tables, and figures.

## 3. Results

Mortality rates of *B. germanica* exposed to six gel bait formulations increased over time (24–120 h) across all populations and developmental stages. However, the univariate ANOVA showed that mortality was significantly affected by population (*F*(3, 720) = 53.76, *p* < 0.001), active ingredient (*F*(5, 720) = 310.03, *p* < 0.001), exposure time (*F*(4, 720) = 74.63, *p* < 0.001), and developmental stage (*F*(2, 720) = 349.84, *p* < 0.001). In addition, analyses revealed that the six tested insecticides differed significantly from each other in terms of mortality (*p* < 0.05). In general, adult females (Duncan test: 77.64%) and males (79.44%) exhibited higher susceptibility compared to nymphs (49.03%).

### 3.1. Imidacloprid

**Figure 3:**
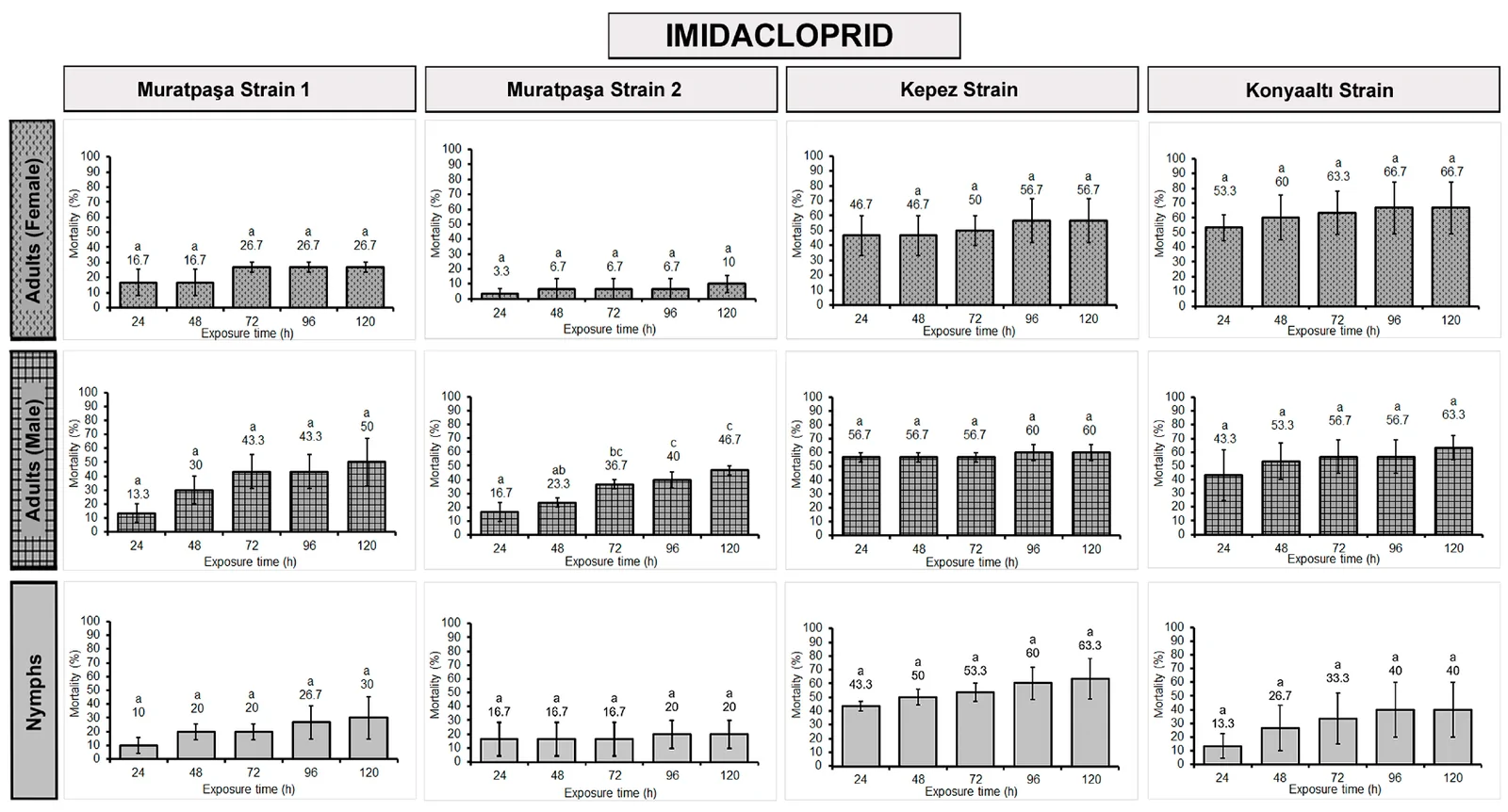
Mortality (%) of *B. germanica* populations exposed to imidacloprid gel bait over a 120 h exposure period. Bars represent mean ± SE. Different lowercase letters indicate significant differences among exposure times within the same field population (strain) for each developmental stage (*p* < 0.05).

Imidacloprid exhibited variable toxicity across the four *B. germanica* populations, with clear differences among life stages and strains. Overall, adults were generally more susceptible than nymphs. The highest susceptibility was observed in the Konyaaltı strain, where mortality in adult females increased from 53.3% at 24 h to 66.7% at 96–120 h, while adult males reached 63.3% mortality at 120 h. In contrast, nymphal mortality in this strain remained lower, not exceeding 40%. Moderate susceptibility was observed in the Kepez strain, with adult mortality ranging between 46.7% and 60.0%, while nymphs showed a gradual increase, reaching 63.3% at 120 h. Lower susceptibility was detected in the Muratpaşa strains, particularly in Muratpaşa strain 2, where adult female mortality remained very low (3.3–10.0%) and nymphal mortality did not exceed 20.0%. Marked differences in susceptibility to imidacloprid were observed among populations and developmental stages (Figure 3).

### 3.2. Indoxacarb

**Figure 4:**
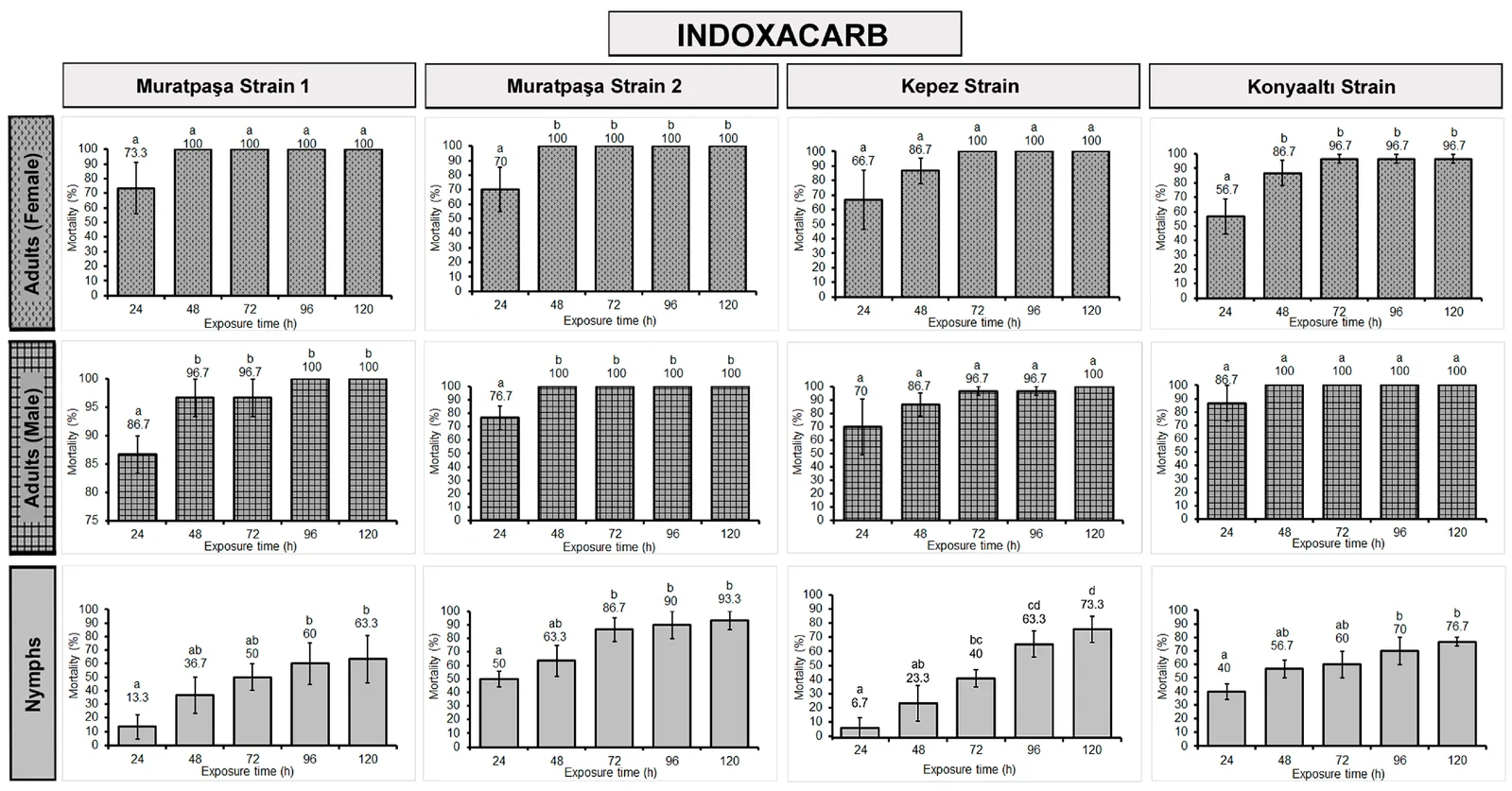
Mortality (%) of *B. germanica* populations exposed to indoxacarb gel bait over a 120 h exposure period. Bars represent mean ± SE. Different lowercase letters indicate significant differences among exposure times within the same field population (strain) for each developmental stage (*p* < 0.05).

Indoxacarb exhibited high toxicity across all tested *B. germanica* populations, with rapid increases in mortality over time. Adult stages were highly susceptible, with mortality reaching 90–100% within 48–72 h in most populations. Rapid adult mortality was observed in all populations, particularly in the Muratpaşa strain 1, Kepez, and Konyaaltı strains, where mortality reached approximately 97–100% within 48–72 h. In contrast, nymphs generally showed a slower and more variable response, although mortality still reached 60–90% by 120 h in most populations. The Konyaaltı strain followed a similar pattern, with complete adult mortality by 72 h, while nymphal mortality remained comparatively lower. Notably, Muratpaşa strain 2 exhibited the highest nymphal susceptibility, with mortality reaching 93.3% at 120 h. Overall, indoxacarb consistently produced high mortality across all populations, particularly in adult stages (Figure 4).

### 3.3. Dinotefuran

**Figure 5:**
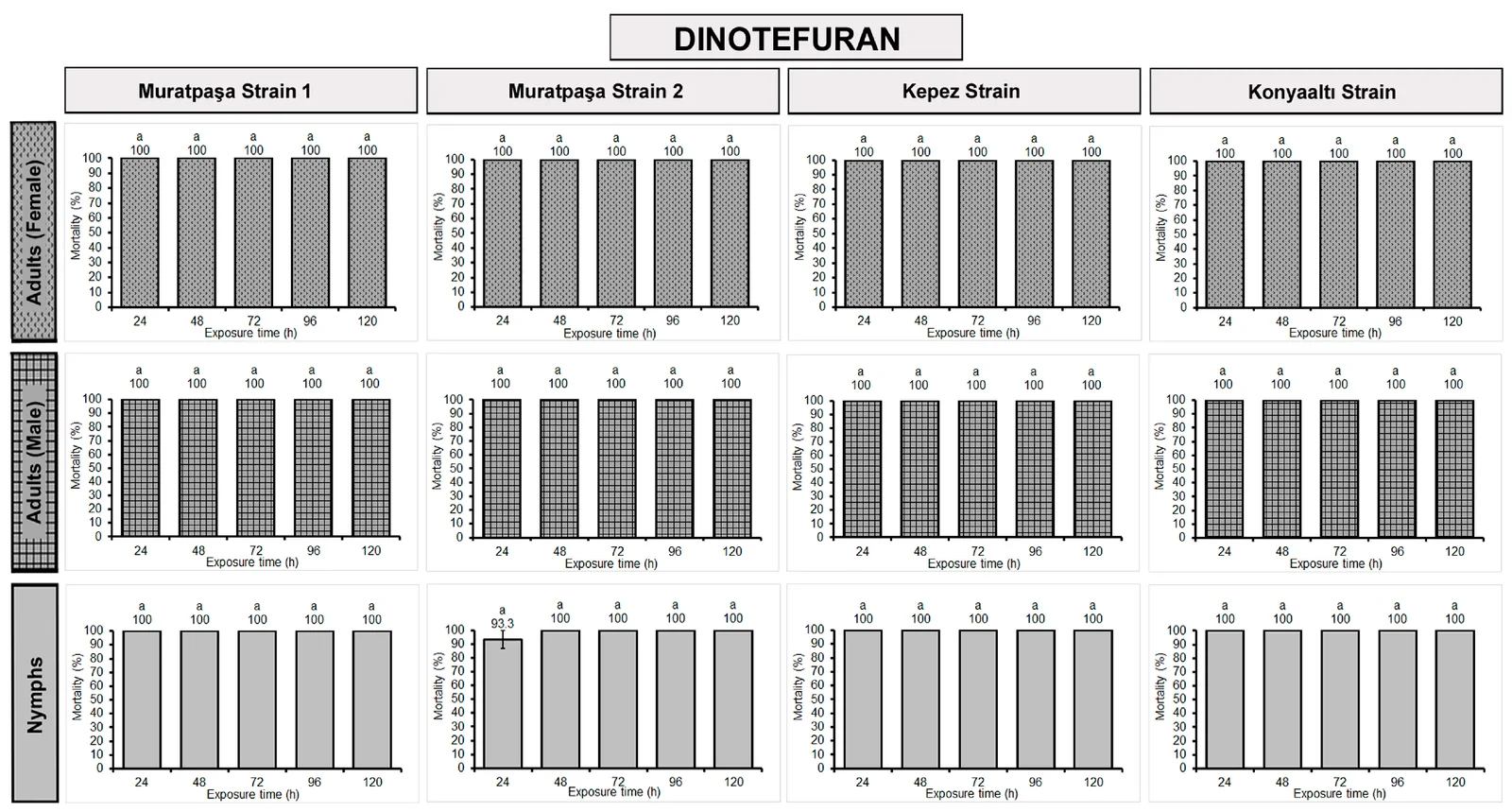
Mortality (%) of *B. germanica* populations exposed to dinotefuran gel bait over a 120 h exposure period. Bars represent mean ± SE. Different lowercase letters indicate significant differences among exposure times within the same field population (strain) for each developmental stage (*p* < 0.05).

Dinotefuran exhibited extremely high and consistent toxicity across all tested *B. germanica* populations and developmental stages. In all populations, adult females and males reached 100% mortality within 24 h. Nymphs also exhibited very high susceptibility. Complete mortality was observed in all populations except Muratpaşa strain 2, where mortality increased from 93.3% at 24 h to 100% at 48 h. Overall, dinotefuran consistently produced rapid and complete mortality across all populations and developmental stages (Figure 5).

### 3.4. Spinosad

**Figure 6:**
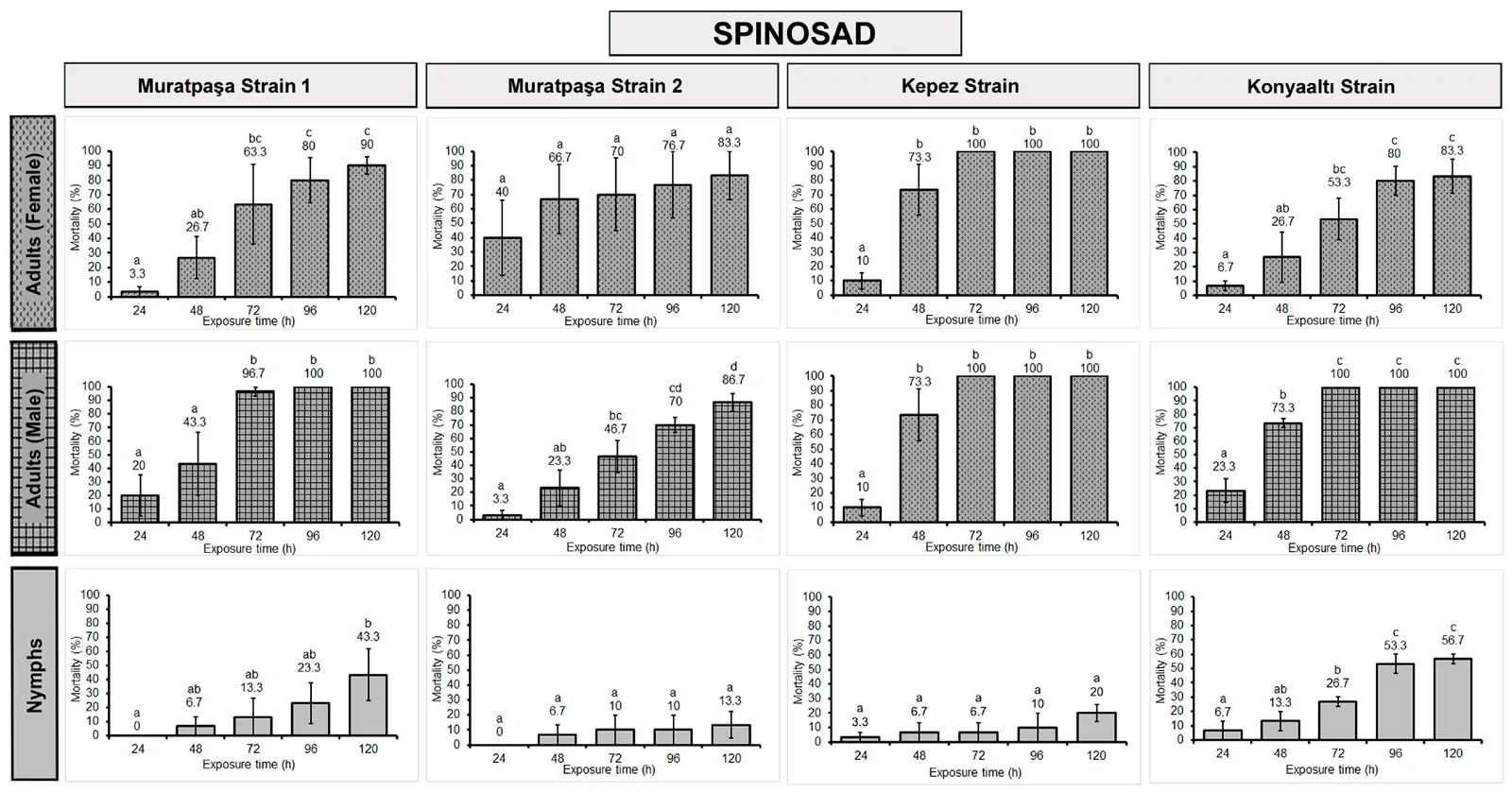
Mortality (%) of *B. germanica* populations exposed to spinosad gel bait over a 120 h exposure period. Bars represent mean ± SE. Different lowercase letters indicate significant differences among exposure times within the same field population (strain) for each developmental stage (*p* < 0.05).

Spinosad exhibited moderate and highly variable toxicity across *B. germanica* populations, with clear differences among developmental stages. Adult stages were generally more susceptible, with mortality reaching up to 100% within 72–96 h in most populations. However, the response was less consistent than that observed for dinotefuran and indoxacarb. In contrast, nymphal mortality remained low to moderate, ranging from 13.3% in Muratpaşa strain 2 to 56.7% in the Konyaaltı strain at 120 h. Marked differences in mortality were observed among developmental stages and populations. Overall, spinosad exhibited moderate efficacy because, although it produced high mortality in adults, mortality in nymphs was considerably lower and more variable (Figure 6).

### 3.5. Clothianidin

**Figure 7:**
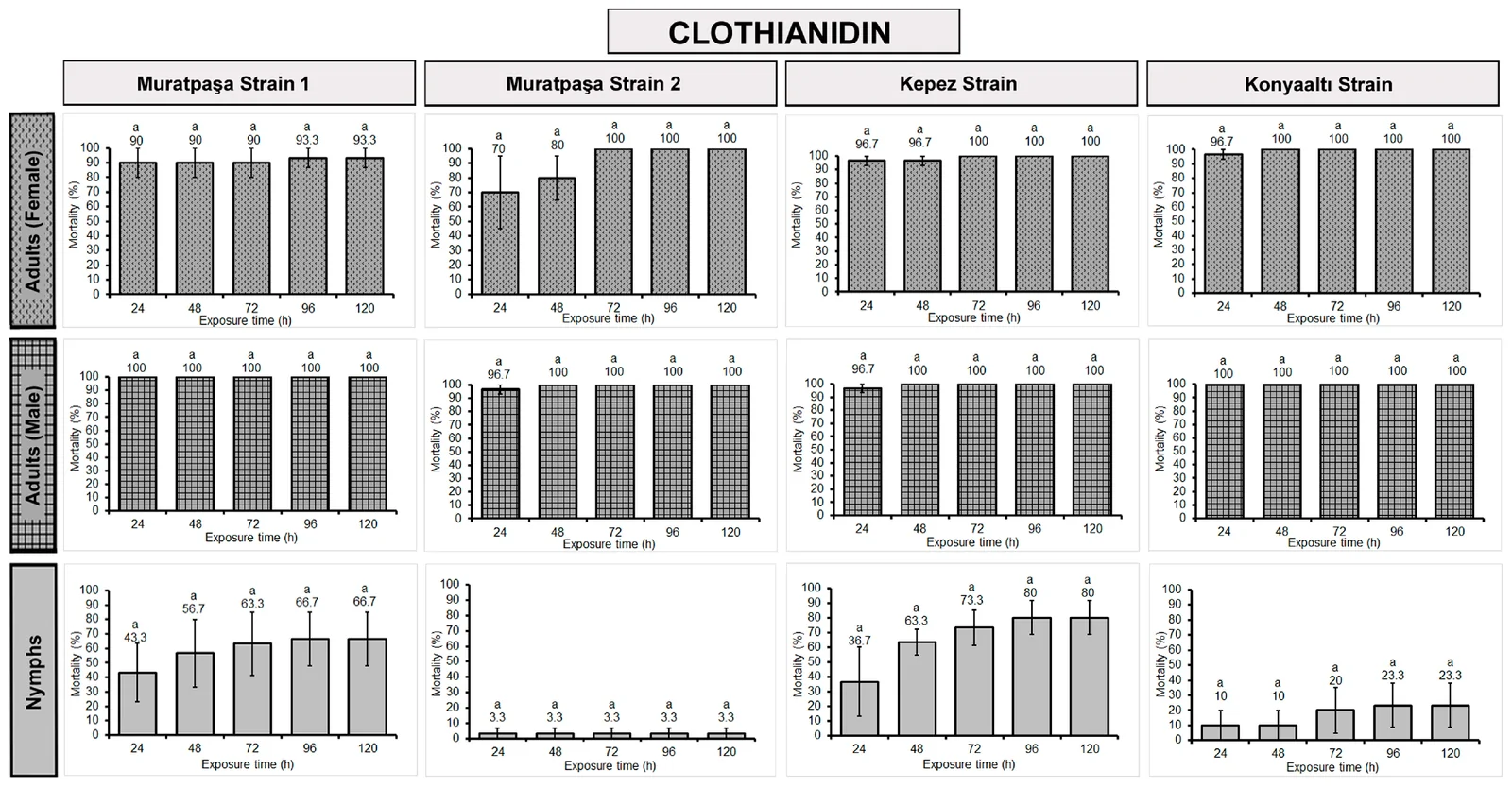
Mortality (%) of *B. germanica* populations exposed to clothianidin gel bait over a 120 h exposure period. Bars represent mean ± SE. Different lowercase letters indicate significant differences among exposure times within the same field population (strain) for each developmental stage (*p* < 0.05).

Clothianidin exhibited high efficacy against adult stages of *B. germanica* across all tested populations, with mortality reaching or approaching 100% within 48–72 h. In contrast, nymphal mortality was consistently lower and more variable than that observed in adults. Nymphal mortality ranged from moderate levels (approximately 60–80%) in the Kepez and Muratpaşa strain 1 populations to very low levels in the Konyaaltı and Muratpaşa strain 2 populations, where mortality remained below 25% and 5%, respectively, even at later time points. These results demonstrate clear stage- and population-dependent differences in mortality following clothianidin exposure, with consistently higher mortality observed in adults than in nymphs (Figure 7).

### 3.6. Fipronil

**Figure 8:**
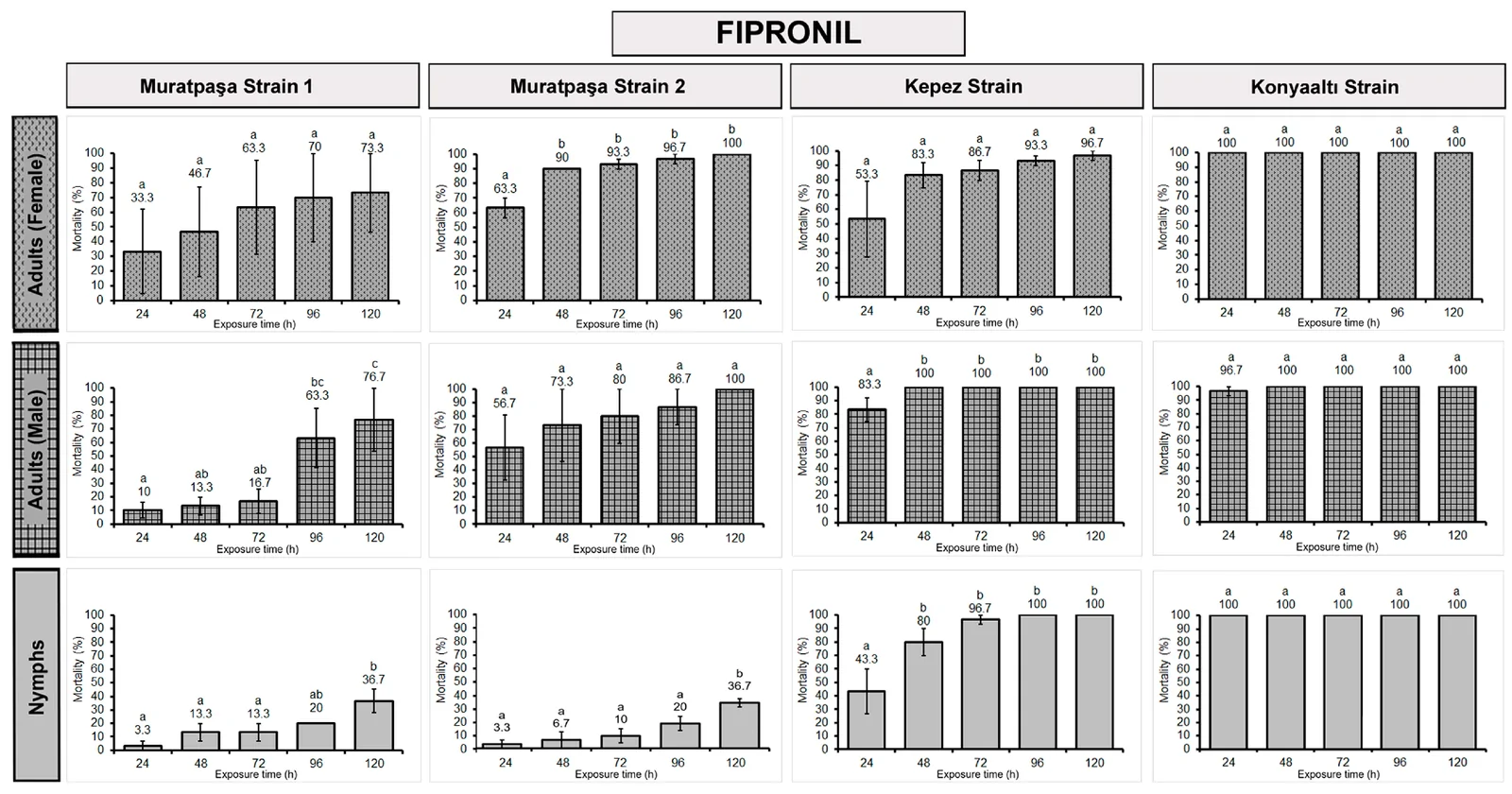
Mortality (%) of *B. germanica* populations exposed to fipronil gel bait over a 120 h exposure period. Bars represent mean ± SE. Different lowercase letters indicate significant differences among exposure times within the same field population (strain) for each developmental stage (*p* < 0.05).

Fipronil exhibited high but variable toxicity across *B. germanica* populations, with clear differences among developmental stages. Adult mortality varied among populations but generally reached high or complete levels (90–100%) by the end of the observation period. In contrast, nymphal mortality showed marked population-dependent variation. Complete mortality was achieved in the Konyaaltı strain throughout the experiment and in the Kepez strain by 96–120 h, whereas nymphal mortality remained below 40% in both Muratpaşa strains. Particularly in Muratpaşa strain 2, nymphal mortality remained very low despite high adult mortality, indicating marked stage-dependent differences in mortality. Overall, fipronil produced consistently high mortality in adults, whereas mortality in nymphs varied considerably among populations (Figure 8).

### 3.7. Comparative Efficacy of Tested Insecticides

When the six tested insecticides were compared, clear differences in efficacy (*F* = 310.03, *p* < 0.001) and population-dependent responses (*F* = 53.76, *p* < 0.001) were observed. Dinotefuran exhibited the highest and most consistent toxicity, producing rapid and complete mortality across all populations and developmental stages (Duncan test: 98.89%, *p* < 0.05) (Figure 5). Indoxacarb also showed very high efficacy (80.67%, *p* < 0.05), particularly in adults, achieving near-complete mortality within 48–72 h, whereas nymphal mortality increased more gradually (Figure 4).

In contrast, imidacloprid demonstrated the lowest mortality across all populations (37.33%, *p* < 0.05), particularly in the Muratpaşa strains (Figure 3). Spinosad showed moderate efficacy (47.11%, *p* < 0.05), producing high mortality in adults but lower and more variable mortality in nymphs (Figure 6). Fipronil (71.17%, *p* < 0.05) and clothianidin (77.06%, *p* < 0.05) exhibited intermediate performance, with high adult mortality but lower and population-dependent mortality in nymphs (Figure 7 and Figure 8).

Among the populations, the Konyaaltı (75.70%, *p* < 0.05) and Kepez strains (75.07%, *p* < 0.05) exhibited the highest overall susceptibility, whereas both Muratpaşa strains showed lower susceptibility (61.96 and 62.07%, *p* < 0.05). However, during the nymphal stage, the Muratpaşa strain 2 generally exhibited the lowest mortality against most of the tested insecticides (Figure 3, Figure 6 and Figure 8). The Kepez strain showed high susceptibility to most insecticides, whereas the Muratpaşa strain 1 exhibited more variable responses depending on both the insecticide and developmental stage.

Overall, these findings demonstrate that insecticide efficacy was strongly influenced by both population origin and developmental stage. Univariate ANOVA showed that developmental stage had a significant effect on mortality (*F*(2, 720) = 349.838, *p* < 0.001). Duncan’s multiple range test showed that mortality in nymphs (49.03%) was significantly lower than that in adult females (77.64%) and adult males (79.44%) (*p* < 0.05), whereas no significant difference was detected between adult females and males (*p* > 0.05).

## 4. Discussion

The control of the German cockroach, *B. germanica*, has become increasingly difficult worldwide due to the widespread development of resistance to multiple classes of insecticides [11,20,21]. Resistance to synthetic pyrethroids, in particular, has been extensively documented and is largely attributed to their long-term and extensive use [10]. Moreover, spray formulations, commonly applied to harborages such as cracks and crevices, may contribute to resistance development by repeatedly exposing cockroach populations to sublethal doses of insecticides.

In line with these findings, the pyrethroid-resistant field populations evaluated in the present study highlight the need for alternative control strategies based on insecticides with different modes of action. The present results demonstrate that gel bait formulations containing neonicotinoids, spinosyns, phenylpyrazole, and oxadiazine provide effective alternatives for managing pyrethroid-resistant *B. germanica* populations, although their efficacy varied according to active ingredient, population, developmental stage, and exposure time.

The univariate ANOVA confirmed that mortality was significantly affected by active ingredient, population, developmental stage, and exposure time (*p* < 0.001). These findings demonstrate that insecticide efficacy depends not only on the chemical properties of the active ingredient but also on the biological characteristics of the target populations. The significant effects of these factors indicate that successful management of pyrethroid-resistant *B. germanica* requires careful consideration of both insecticide selection and population characteristics.

The significant effect of the active ingredients observed in the univariate analysis was reflected in the markedly different mortality patterns among the tested gel bait formulations (*p* < 0.05). Dinotefuran consistently produced the highest mortality, whereas imidacloprid exhibited the lowest efficacy across most populations, indicating substantial differences in insecticidal performance among compounds. Population also markedly influenced mortality, with the Muratpaşa populations generally exhibiting lower mortality than the Konyaaltı population, consistent with population-specific differences in resistance profiles, as similarly reported by Oz et al. [10]. Exposure time further affected mortality, with most gel bait formulations producing progressively higher mortality over time, as bait toxicants require ingestion before intoxication and death occur [22,23].

Developmental stage strongly influenced mortality, with adults consistently exhibiting higher susceptibility than nymphs. The lower mortality observed in nymphs may be associated with developmental differences in physiology, reduced bait consumption, and enhanced metabolic detoxification. Increased activity of cytochrome P450 monooxygenases, esterases, and glutathione S-transferases has been reported as one of the mechanisms contributing to insecticide resistance in *B. germanica* [11,24,25]. However, these factors were not directly investigated in the present study.

Among the neonicotinoids evaluated, dinotefuran exhibited the highest efficacy, followed by clothianidin, whereas imidacloprid showed the lowest toxicity. The reduced performance of imidacloprid may reflect differences in susceptibility among the tested field populations and could be associated with resistance or cross-resistance resulting from previous insecticide exposure. However, resistance mechanisms and resistance ratios were not investigated in the present study. Although neonicotinoids share a common mode of action by targeting nicotinic acetylcholine receptors (nAChRs), differences in receptor-binding affinity, receptor subtype selectivity, and target-site interactions may result in considerable variation in biological activity among compounds within this insecticide class [26]. Dinotefuran has been shown to bind with high affinity to insect nAChRs, and receptor-binding affinity has been positively correlated with insecticidal activity in cockroaches [27]. Therefore, the superior efficacy of dinotefuran observed in the present study may partly be explained by its reported higher binding affinity for insect nAChRs. However, receptor-binding affinity was not investigated in the present study, and this interpretation should be considered a possible explanation based on previous studies. This efficacy pattern is generally consistent with previous evaluations of commercial gel bait formulations. Hamilton et al. [28] reported complete or near-complete mortality of *B. germanica* exposed to dinotefuran, clothianidin, and fipronil-based gel baits, whereas Gordon et al. [29] also reported high laboratory efficacy of several commercial gel bait formulations while emphasizing that field performance may vary depending on environmental and operational conditions.

Indoxacarb also demonstrated high efficacy across all tested populations, although its insecticidal activity developed more slowly than that of the other compounds. This pattern is consistent with its mode of action as a pro-insecticide requiring metabolic activation within the insect to form its bioactive metabolite, which subsequently blocks voltage-gated sodium channels, resulting in paralysis and death [30,31]. The requirement for metabolic activation most likely explains the delayed mortality observed during the early exposure periods. Our findings agree with those of Appel [30], who demonstrated excellent laboratory and field performance of indoxacarb gel bait against German cockroach populations despite its relatively slower onset of action.

In contrast, spinosad exhibited moderate and variable efficacy, producing high mortality in adults but substantially lower and more variable mortality in nymphs. The lower susceptibility observed in nymphs may be associated with developmental differences in physiology and metabolic detoxification [11,24,25]. Similarly, clothianidin and fipronil produced consistently high mortality in adults but lower efficacy in nymphs, further supporting pronounced developmental stage-dependent differences in susceptibility. Previous studies have likewise reported that the efficacy of gel bait formulations varies considerably among field populations depending on insecticide susceptibility and resistance status [16,26], supporting the population-dependent responses observed in the present study.

An additional advantage of gel bait formulations reported in previous studies is their ability to promote secondary (horizontal) transfer of insecticides within cockroach populations, thereby increasing mortality among individuals that do not directly consume the bait [28,32]. However, horizontal transfer was not investigated in the present study. This phenomenon may contribute to improved population suppression under field conditions and represents an important advantage of bait-based control strategies over conventional residual spray applications.

It is also important to consider the regulatory and environmental context of these active ingredients. Several neonicotinoids, including imidacloprid and clothianidin, have been prohibited for outdoor agricultural use in the European Union because of concerns regarding their adverse effects on non-target organisms, particularly pollinators. Nevertheless, the present findings demonstrate that these active ingredients remain highly effective against *B. germanica* when delivered as gel bait formulations in indoor environments. Previous studies have suggested that indoor gel bait applications are expected to reduce exposure of pollinators and other non-target organisms compared with outdoor agricultural uses [33,34,35,36]. However, non-target exposure was not evaluated in the present study.

Unlike conventional spray applications, gel baits provide a more targeted approach to German cockroach control by delivering small quantities of insecticide directly to harborages and foraging sites [13]. Consequently, gel baits minimize insecticide dispersion, environmental contamination, non-target exposure, and potential human health risks. In contrast, residual spray applications may result in widespread insecticide deposition, increasing the likelihood of inhalation, dermal exposure, and contamination of indoor environments. Gordon et al. [29] further demonstrated that although gel bait formulations generally exhibit high efficacy under laboratory conditions, their performance in residential environments may be influenced by bait placement, alternative food sources, harborage availability, and environmental complexity. Therefore, laboratory efficacy should be interpreted together with field conditions when selecting gel bait formulations for practical cockroach management. From both environmental and public health perspectives, gel bait formulations represent a safer, more targeted, and more sustainable approach for German cockroach management.

Overall, the results of this study demonstrate that insecticide efficacy is strongly influenced by both population-specific resistance patterns and developmental stage. The superior performance of dinotefuran, together with the consistently high efficacy of indoxacarb, highlights the importance of incorporating alternative insecticides with different modes of action into integrated pest management programs. Furthermore, the reduced susceptibility observed in certain populations, particularly during the nymphal stage, emphasizes the importance of continuous resistance monitoring and careful insecticide selection. The findings of the present study, together with previous evaluations of commercial gel bait formulations, indicate that insecticides with newer modes of action remain highly effective against many pyrethroid-resistant populations. Rotating gel bait formulations containing insecticides with different modes of action may help delay the evolution of resistance and prolong the effectiveness of cockroach management programs, thereby contributing to more effective and sustainable control of *B. germanica* [11,37].

The present study has some limitations that should be considered when interpreting the findings. The evaluated products were commercial gel bait formulations rather than technical-grade active ingredients. Therefore, differences in efficacy may have been influenced not only by the active ingredients but also by formulation characteristics, including bait matrix, attractants, and other co-formulants. In addition, the concentrations of the active ingredients differed among the commercial formulations evaluated. Consequently, the observed differences in efficacy should be interpreted as reflecting the overall performance of the tested gel bait formulations rather than the intrinsic toxicity of the active ingredients alone.

## 5. Conclusions

The present study demonstrated that the efficacy of gel bait insecticides against pyrethroid-resistant *B. germanica* populations varies significantly according to active ingredient, population origin, developmental stage, and exposure time. Univariate ANOVA confirmed that all these factors significantly influenced mortality, highlighting the complexity of insecticide performance under resistance conditions.

Among the six active ingredients evaluated, dinotefuran exhibited the highest and most consistent efficacy across all populations and developmental stages, achieving rapid and nearly complete mortality even in pyrethroid-resistant field populations. Indoxacarb also provided excellent control, although its insecticidal activity developed more gradually because of its requirement for metabolic activation. In contrast, imidacloprid showed the lowest efficacy, suggesting possible reduced susceptibility or cross-resistance in some field populations. Clothianidin, spinosad, and fipronil produced intermediate but population- and stage-dependent responses, with nymphs consistently exhibiting lower susceptibility than adults.

These findings emphasize that developmental stage should be considered when selecting gel bait formulations because immature stages may survive treatments that effectively eliminate adults. Likewise, the observed differences among field populations demonstrate the importance of local resistance profiles when designing cockroach management programs.

A limitation of the present study is that resistance ratios, LC_50_ values, and molecular or biochemical resistance mechanisms were not evaluated. Future studies incorporating these approaches would provide a more comprehensive characterization of the resistance status of the tested populations.

Beyond their high efficacy, gel bait formulations offer important operational advantages, including targeted delivery, reduced insecticide dispersion, lower risks to humans and non-target organisms, and the potential for horizontal transfer within cockroach populations. Together, these characteristics make gel baits valuable components of integrated pest management programs.

To the best of our knowledge, this study represents the first comprehensive comparative evaluation of six commercially available gel bait formulations with different modes of action against pyrethroid-resistant field populations of *B. germanica* in Türkiye. The results support the continued use and rotation of gel bait formulations with different modes of action as an effective strategy for delaying resistance development and improving the long-term sustainability of German cockroach control. Future field-based studies conducted under operational conditions will further improve our understanding of gel bait performance and optimize resistance management strategies.

## Figures and Tables

**Table 1:** Characteristics of the gel bait formulations used in the study.

Active Ingredient	Chemical Class	CAS Number	Concentration (%)	Mode of Action (IRAC Group)
Clothianidin	Neonicotinoid	210880-92-5	2	Nicotinic acetylcholine receptor agonist (4A)
Dinotefuran	Neonicotinoid	165252-70-0	2	Nicotinic acetylcholine receptor agonist (4A)
Fipronil	Phenylpyrazole	120068-37-3	0.05	GABA-gated chloride channel blocker (2B)
Imidacloprid	Neonicotinoid	138261-41-3	2.15	Nicotinic acetylcholine receptor agonist (4A)
Indoxacarb	Oxadiazine	144171-61-9	0.06	Voltage-gated sodium channel blocker (22A)
Spinosad	Spinosyn	168316-95-8	2	Nicotinic acetylcholine receptor allosteric activator (5)

## Data Availability

The raw mortality data and replicate values supporting the findings of this study are provided in Supplementary Dataset S1. Additional information is available from the corresponding author upon reasonable request.

## Supplementary Materials

The following supporting information can be downloaded at: https://www.mdpi.com/article/10.3390/pathogens15080842/s1, Supplementary Dataset S1: Raw mortality data and replicate values.

## Abbreviations

The following abbreviations are used in this manuscript:

±	Plus–minus
%	Percent
<	Less than
ANOVA	Analysis of Variance
CAS	Chemical Abstracts Service
cm	Centimeter
cm^2^	Square Centimeters
°C	Degree Celsius
e.g.,	Exempli Gratia (For Example)
*F*	F distribution
g	Gram
h	Hour
IBM	International Business Machines
IRAC	Insecticide Resistance Action Committee
nAChRs	Nicotinic Acetylcholine Receptors
*p*	Probability
P450	Cytochrome P450 monooxygenase
SE	Standard Error
SPSS	Statistical Package for the Social Sciences
